# Glucagon-like peptide-1 agonist use for obesity treatment in patients with left ventricular assist devices

**DOI:** 10.1016/j.jhlto.2024.100114

**Published:** 2024-05-28

**Authors:** Michele Edwards, Melanie Thomas, Maryjane Farr, Densey Varghese, Lauren K. Truby, Jennifer T. Thibodeau, Mark H. Drazner, Matthias Peltz, Justin L. Grodin, Nicholas S. Hendren

**Affiliations:** a Department of Internal Medicine, UT-Southwestern, Dallas, Texas; b Department of Cardiothoracic Surgery, UT-Southwestern, Dallas, Texas; c Parkland Health, Dallas, Texas

**Keywords:** LVAD, obesity, GLP-1a, heart failure, heart transplant

## Abstract

Glucagon-like peptide-1 agonists are effective weight loss treatments, yet few data are available regarding their use in patients with durable left ventricular assist devices. We report our single-center experience with glucagon-like peptide-1 agonist use in 21 patients with World Health Organization class I to III obesity on durable left ventricular assist device support. During therapy, patients experienced a median weight change of −12.4 (−20.0 to −2.9) kg and a −7.4% (−16.3 to −2.1) change in body weight with a median time on glucagon-like peptide-1 agonist therapy of 12 (6-22) months. Of these, 17 of 21 patients lost weight (median change −14.0kg [−21.8 to −5.3], −9.5% [−18.0 to −4.0] body weight) and 9 of 21 had a speed reduction. No major adverse events were attributed to glucagon-like peptide-1 agonist therapy. At our center, glucagon-like peptide-1 agonists use was well tolerated, safe, and associated with weight loss in patients with durable left ventricular assist devices.

Durable left ventricular assist devices (LVAD) have been implanted in >20,000 patients with end-stage heart failure.^1^ Obesity is a common comorbid condition in LVAD recipients and a body mass index (BMI) >35 kg/m^2^ is considered an absolute or relative contraindication for heart transplantation (HT). Glucagon-like peptide-1 agonists (GLP-1a) are an effective weight loss treatment for patients with obesity and heart failure.^2^, ^3^ Whether GLP-1a is associated with weight loss in patients with obesity and LVAD and/or influences LVAD parameters is unknown. Herein, we report our single-center experience with GLP-1a use in patients with obesity and durable LVADs.

Our study conforms with the International Soceity for Heart and Lung Transplantation ethics guidance. Following institutional review board approval, we abstracted data from the electronic health record for all patients implanted with durable LVAD between January 2020 and October 2023 (N = 80) at UT Southwestern Medical Center. We identified all patients implanted with an LVAD and BMI >30 kg/m^2^ (N = 21) who were initiated on a GLP-1a postimplant. Next, we determined the median changes in weight, HT candidacy, natriuretic peptides, LVAD settings, and medical management from the initiation of GLP-1a therapy to the end of follow-up (all remained on GLP-1a therapy). Continuous variables are reported as median (interquartile range (IQR)) and categorial as number (%).

There were 21 patients implanted with an LVAD with BMI >30 kg/m^2^ and who were prescribed GLP-1a after LVAD implantation and 18 of 21 (86%) had a baseline BMI >35 kg/m^2^. Baseline characteristics are shown in Table 1. Fifteen of 21 (71%) patients had obesity listed as a relative or absolute contraindication to HT at the time of LVAD implantation. One patient had a HeartMate 2, 14 a HeartMate 3, and 6 a HeartWare LVAD. The median time on GLP-1a therapy was 12 (6-22) months. Patients experienced a median weight change of −12.4 (−20.0 to −2.4) kg and a −3.7 (−7.6 to −0.7) kg/m^2^ change in BMI (Figure 1) which corresponded to a −7.4% (−16.3 to −2.1) change in body weight. While on GLP-1a therapy, 17 of 21 patients had net weight loss and of the patients who lost weight, 9 (53%) had a net LVAD speed reduction on GLP-1a therapy. The primary reason for LVAD speed reduction was arrhythmia (*n* = 1), echocardiographic findings (e.g., small left ventricle size, etc.) (*n* = 3), and suction waveform and pulsatility index events with speed drops (*n* = 5). For patients with a baseline BMI >35 kg/m^2^ (N = 18), 6 of 18 (33%) patients achieved BMI <35 kg/m^2^ after initiation of GLP-1a, with 3 undergoing HT evaluation and 2 listed for HT. While on GLP-1a therapy, 10 of 21 patients had diuretic doses lowered. N-terminal pro b-type natriuretic peptide levels (−91 [−454 to 91] pg/ml) were also observed to be lower. Fourteen LVAD recipients had available pre- and post-GLP-1a six minute walk test data available with an increase of 53 (32-69) meters from a baseline of 262 (149-319) meters. No serious adverse events attributed to GLP-1a were observed. While on GLP-1a, 9 of 21 patients developed a new driveline infection after initiation of GLP-1a use while 6 of 21 patients did not have a driveline infection (pre- or post-GLP-1a use) and 6 of 21 patients had a driveline infection that predated GLP-1a use. One patient underwent bariatric surgery after several months on GLP-1a therapy with associated significant weight loss prebariatric surgery.

**Table 1:** Baseline Characteristics

Variable	Patient with an LVAD and GLP-1a prescription (*n* = 21)
Age, years	45 (42-52)
Male	16 (76)
Race/ethnicity	
Non-Hispanic Black	13 (61)
Non-Hispanic White	2 (10)
Hispanic White	3 (14)
Asian	0 (0)
Other	3 (14)
Months on LVAD therapy before GLP-1a	9 (3-17)
Device type	
HVAD	6 (29)
HeartMate II	1 (4)
HeartMate III	14 (67)
LVAD strategy—destination therapy	21 (100)
Reasons for strategy	
Body mass index	15 (71)
Medication adherence	2 (10)
Substance abuse	2 (10)
Other	2 (10)
LVAD speed reduction	9 (43%)
Suction waveform/speed drop/PI events	5 (24)
Echocardiogram findings	3 (14)
Arrhythmia	1 (5)
GLP-1a agent	
Dulaglutide, weekly injection	1 (5)
Semaglutide, weekly injection	18 (86)
Semaglutide, oral tablet	1 (5)
Tirzepatide, weekly injection	1 (5)
Comorbid medical conditions	
Diabetes	13 (62)
Hypertension	19 (60)
Ischemic cardiomyopathy	3 (14)
Prior stroke	6 (29)

Abbreviations: GLP-1a, glucagon-like peptide-1 agonists; HVAD, HeartWare; LVAD, left ventricular assist devices; PI, pulsatility index.

Continuous variables are median (IQR) and categorical variables are N (%).

**Figure 1:**
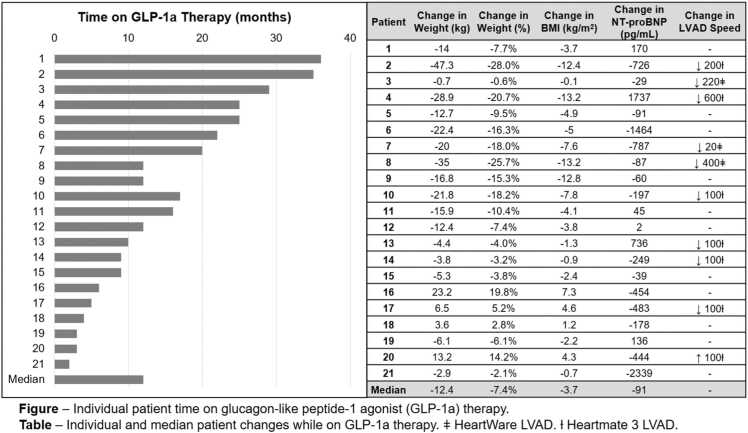
Individual and median patient time on GLP-1a therapy and changes in weight, body mass index, and LVAD speed changes. BMI, body mass index; GLP-1a, glucagon-like peptide-1 agonists; LVAD, left ventricular assist devices.

Obesity is strongly associated with the risk for heart failure hospitalization and World Health Organization class II or greater obesity (BMI >35 kg/m^2^) is frequently a relative contraindication to HT for patients with end-stage heart failure.^4^ Multiple studies have shown that GLP-1a therapy is highly effective at promoting substantial weight loss within several months and reducing the risk of cardiovascular death or heart failure hospitalization in patients with heart failure.^2^, ^3^ Indeed, most patients in our cohort lost weight on GLP-1a therapy, and of those who lost weight, 8 of 17 (47%) lost >15% body weight. Importantly, one-third (6 of 18) of patients with World Health Organization class II and III obesity lost enough weight to become HT candidates with a BMI of ≤35 kg/m^2^.^4^ We observed that patients who lost weight frequently had LVAD speed reductions, highlighting the need for careful clinical monitoring and consideration of echocardiographic surveillance to guide clinical LVAD care during periods of substantial weight loss. Rapid weight loss due to less caloric intake may induce a reduction in diuretic requirements and blood pressure which theoretically could result in adverse events for patients on high doses of guideline directed medical therapy for heart failure. It is not clear that GLP-1a directly led to speed reduction; however, during GLP-1a therapy we observed lower natriuretic peptides and diuretic requirements. Our findings are inherently limited by the single-arm retrospective observational nature of the study design and future studies with larger cohorts are needed. Importantly, despite GLP-1a initiation, we did not observe any clinically serious adverse events attributed to GLP-1a use. In summary, in a single-center, observational cohort, GLP-1a use in patients with LVADs was well tolerated, safe, and frequently promoted significant weight. For patients with a BMI contraindication to HT, GLP-1a use may promote substantial weight loss and resolve weight as a barrier to HT.

## Author contributions

All authors provided substantial contributions via project design, data analysis, and/or interpretation of the data. All authors provided critical review for intellectual content, approved the final version, and are accountable for the accuracy and integrity of the data.

## Disclosure statement

L.K.T. has received funding from the American Heart Association (23CDA1050881) and the UT Southwestern Presidential Research Council. M.F. is a member of the steering committee for TransMedics and is a member of the UNOS/OPTN Board of Directors. N.S.H. reports research grant support from TriCog Health and the American Heart Association; and consulting services from and Tosoh Inc. J.L.G. reports research funding from the Texas Health Resources Clinical Scholarship, Eidos/BridgeBio, Pfizer, and NHLBI R01HL160892. J.L.G. reports consulting fees from Pfizer, Eidos/BridgeBio, Alnylam, Astra-Zeneca, Alexion, and Sarepta. The remaining authors have reported no disclosures.

Acknowledgments and Funding: None.
